# Evaluation of schwannoma using the 3D-SPACE sequence: comparison with the 3D-CISS sequence in 3T-MRI

**DOI:** 10.3906/sag-2010-30

**Published:** 2021-06-28

**Authors:** Enes GÜRÜN, İsmail AKDULUM, Pınar KILIÇ, Nil TOKGÖZ, Murat UÇAR

**Affiliations:** 1 Department of Radiology, İskilip Atıf Hoca State Hospital, Çorum Turkey; 2 Department of Radiology, Gazi University Hospital, Ankara Turkey; 3 Department of Radiology, Pursaklar State Hospital, Ankara Turkey

**Keywords:** Magnetic resonance imaging, 3D-SPACE, 3D-CISS, cerebellopontine angle, schwannoma

## Abstract

**Background/aim:**

The purpose of this study is to compare the diagnostic accuracy and interobserver reliability of the T2-weighted 3D-SPACE (three-dimensional sampling perfection with application-optimized contrasts by using different flip angle evolutions) sequence in comparison with T2-weighted 3D-CISS (three-dimensional constructive interference in steady state) sequences for diagnosis of schwannomas.

**Materials and methods:**

Forty patients with cerebellopontine angle (CPA), internal acoustic canal (IAC), and cochlear schwannoma who had undergone magnetic resonance imaging (MRI) using the 3D-CISS and 3D-SPACE sequences were identified. The sequences were retrospectively evaluated by two radiologists for the qualitative analyses, which were subsequently compared using the Mann–Whitney U test. Following this, kappa values were used for interobserver agreement. P < 0.05 was considered to be of statistical significance.

**Results:**

The interobserver agreement was found to be excellent between the two observers for the interpretation of all qualitative analyses for both sequences (kappa value > 0.8). The 3D-SPACE sequences demonstrated significantly better qualitative scores and fewer artifacts compared with the 3D-CISS sequences (p < 0.05).

**Conclusion:**

Our results demonstrate that 3D-SPACE is superior to 3D-CISS in the imaging process of the schwannoma in terms of image quality, description of the relationship between the lesion and cranial nerve, signal differentiation between lesion and cistern, and signal differentiation between the lesion and adjacent brain.

## 1. Introduction

Schwann cells are responsible for producing the myelin sheath, such as oligodendrocytes of the central nervous system [1]. A schwannoma is a benign tumor that originates from Schwann cells in the neural crest; it was discovered by Theodor Schwann [2]. Schwannomas of the cranial nerves involving the trigeminal, facial, and vestibular nerve may be limited in the internal acoustic canal (IAC) or extend into the cerebellopontine angle (CPA). Schwannomas are the most common benign brain tumors, accounting for six to eight percent of all intracranial tumors and approximately 80% of all CPA tumors [3]. Meningiomas are the second most common benign CPA tumors after schwannomas [4]. 

The modalities needed for CPA, IAC, and cochlear schwannoma imaging are computed tomography (CT) and magnetic resonance imaging (MRI). MRI allows for excellent soft-tissue resolution and multiplanar examination, making it the first modality used to evaluate CPA schwannomas. The shape and margin characteristics, extension, mass effect, internal structure characteristics, and the relationship between adjacent soft tissues can be evaluated with MRI [5].Thus, MRI can be used for tumor characterization, surgical planning, and postoperative follow-up. Contrast-enhanced temporal bone CT may be performed in patients with contraindications for MRI. 

Conventional 2D spin-echo MRI sequences do not provide detailed anatomical information in evaluating CPA. The internal structure, morphology, cistern, and cranial nerve relationship of CPA schwannomas can be better demonstrated by new 3D pulse sequences compared to conventional MRI examinations. Moreover, three-dimensional constructive interference in steady-state (3D-CISS) and three-dimensional sampling perfection with application-optimized contrasts by using different flip angle evolutions (3D-SPACE) sequences provide isotropic information and thinner images in any plane. 3D-SPACE is a T2-weighted 3D turbo spin-echo sequence using a variable flip angle instead of conventional 180° pulse sequences [6,7]. Thin sections result in better spatial resolution and reduced partial volume artifact. These sequences allow us to evaluate the relationship between millimeter lesions and adjacent tissue [8]. Contrast-enhanced 3D-SPACE imaging shows the signals of the different components in T2-weighted imaging and the difference in contrast between the lesion and the cranial nerve. 3D-CISS is a type of steady-state sequence based on a fast gradient-echo sequence [9]. In 3D-CISS, the image contrast is determined by the T2 / T1 tissue ratio. Tissues with long T2 relaxation and short T1 relaxation times have a high signal intensity. Therefore, tissues with a high T2 / T1 ratio, such as water and fat, produce a high signal in this sequence. This provides excellent contrast between cerebrospinal fluid (CSF) and other structures [10]. With all of these, 3D-CISS has an important role with high signal to noise and contrast to noise ratios in evaluating tissues surrounded by cerebrospinal fluid. Total resection or functional protection may be preferred in the management of CPA schwannomas. Functional protection may be preferred when the cranial nerve cannot be fully preserved, and the lesion cannot be safely resected [11].

This study aimed to compare the diagnostic accuracy and interobserver reliability of the T2-weighted 3D-SPACE sequence with the T2-weighted 3D-CISS sequences for the diagnosis of CPA, IAC, and cochlear schwannoma.

## 2. Materials and methods

### 2.1. Patients

Forty patients with CPA, IAC, and cochlear schwannoma who had undergone MRI using the 3D-CISS and 3D-SPACE sequences were identified retrospectively. The study population included 40 patients who had been diagnosed with schwannoma. One patient with diffuse cystic lesion was excluded from the study as an exclusion criterion. The patients’ ages were in the range of 34–61 years. None of the patients had previously undergone surgery for CPA since patients without a pathological diagnosis were included in our study, and the radiological diagnosis was based on findings such as the enlargement of porus acusticus and the absence of dural tail [12]. The retrospective study was approved by our Institutional Review Board (38-13.01.2020). Informed consent was waived due to the retrospective nature of this study.

### 2.2. MRI protocol

All studies were performed with an 8-channel head coil and a 3.0-T MRI system (Verio; Siemens Medical Solutions, Erlangen, Germany). MRI sequences included axial T2-weighted FSE sequences, 3D-SPACE, 3D-CISS sequences, pre-contrast axial, coronal T1-weighted FSE sequences and postcontrast axial, and coronal T1-weighted FSE sequences. Postcontrast images were obtained by injecting 20 cc of gadoteric acid (Gd-DOTA, Dotarem) injection at a rate of 5 mL/s through a peripheral arm vein. Both 3D-SPACE and CISS sequences were used in the same patient to assess the success in qualitative evaluation in both sequences. 3D-SPACE and 3D-CISS sequences were obtained using the following parameters: repetition time (TR) 3200 ms; time to echo (TE) 402 ms; the number of excitations (NEX) 2; the field of view (FOV) 210 mm; 380 x 384 matrix, voxel size 0.8 × 0.8 × 0.8 mm; and parallel imaging technique with an acceleration factor of 2 for 3D-SPACE. The acquisition time was 4 min 18 s. TR/TE = 7.91/3.66 ms; FA 50; FOV 206 mm, voxel size 0.7 × 0.7 × 0.7 mm and 320 x 320 matrix for 3D-CISS. The acquisition time was 3 min 57 s. The 3D CISS and 3D SPACE sequences were performed in the same session. The details of the two sequences are summarized in Table 1.

**Table 1:** Sequence parameters for 3D-SPACE and 3D-CISS (SPACE: sampling perfection with application optimized contrast with different flip-angle evolutions, CISS: constructive interference in the steady-state, 2D: two-dimensional, 3D: three dimensional)

	3D-SPACE	3D-CISS
Repetition time (ms)	3200	7.91
Echo time (ms)	402	3.66
Flip angle (deg)	*	50˚
Bandwidth (Hz/ pixel)	751	460
Echo space (ms)	3.88	x
Average	2	1
Field of view (mm)	206	210
In-plane voxel size (mm)	0.8*0.8*0.8	0.7*0.7*0.7
Acquisition time	4 min 18 s	3 min 57 s

### 2.3. MRI evaluation

Sequences (3D-SPACE and 3D-CISS) were retrospectively evaluated by two radiologists (EG, PK; both radiologists have 5 years of experience in neuroradiology) for the image quality, visibility of the relationship between the schwannoma and cranial nerves, signal differentiation between lesion and cistern, signal differentiation between the lesion and adjacent brain parenchyma, and the severity of artifacts (susceptibility and vessel flow artifacts). The observers were blinded to the imaging and all acquisition parameters.

Using these criteria/parameters we created a 5-point scale system numbering from zero to four under the following assumptions: excellent, 4; good, 3; fair, 2; poor, 1; non-diagnostic, 0.

### 2.4. Statistical analysis

Statistical analyses were performed using SPSS version 22.0 (IBM Corp., Armonk, NY,USA). Data were expressed as a mean with the uncertainty taken to be the standard deviation (SD). Qualitative image analyses were compared using the Mann–Whitney U-test, and kappa values were used for inter-observer agreement. A k value of 0.01 to 0.20 indicates slight agreement; 0.21 to 0.4 means fair agreement; 0.41 to 0.6 indicates moderate agreement; 0.61 to 0.8 demonstrates good agreement; and 0.81 to 1.00 means excellent agreement. P < 0.05 was considered to be statistically significant.

## 3. Results 

The study population included 40 patients who had been diagnosed with schwannoma. 23 patients were female, and the remaining were male. The patient ages ranged from 34 to 61 years and the mean age was 47.2 ± 4.42. The demographic characteristics of the participants are presented in Table 2. 

**Table 2:** The demographic characteristics of the participants.

Variables	Patients (n = 40)
Age (range)	47.2 ± 4.42 (34–61 years)
Sex (male:female)	17:23
BMI (kg/m2)	26.17 ± 4.25

Excellent inter-observer agreement was found between the two observers for the interpretation of all qualitative analysis for both sequences (kappa value > 0.8) (Table 3).

**Table 3:** Kappa statistic for interobserver agreement between two different sequences for qualitative variables (SPACE: sampling perfection with application-optimized contrast with different flip-angle evolutions, CISS: constructive interference in the steady-state, CN: cranial nerve).

Qualitative variables	3D-SPACE	3D-CISS
Overall image quality	0.811 (0.701–0.921) *	0.851 (0.754–0.949) *
Visibility of the relationship between the schwannoma and CN	0.818 (0.715–0.923) *	0.816 (0.709–0.923) *
Signal differentiation between lesion and cistern	0.842 (0.722–0.962) *	0.809 (0.707–0.911) *
Signal differentiation between the lesion and adjacent brain parenchyma	0.814 (0.713–0.915)	0.825 (0.715–0.935) *
Artifacts	0. 822 (0.699–0.945) *	0.805 (0.702–0.908) *

**Figure 1:**
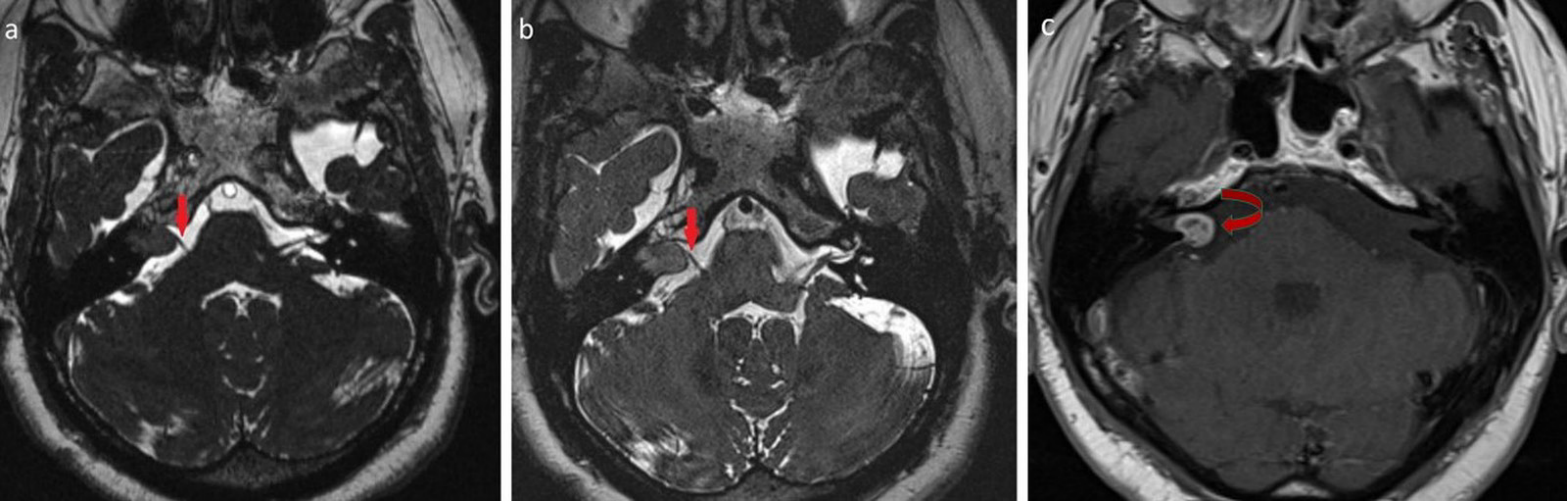
Axial T2-weighted 3D-CISS (a) and 3D-SPACE (b) images of vestibular schwannoma within the right internal auditory canal with extracanalicular extension. The lesion is clearly visualized with each sequence; however, the visibility of the relationship between the mass and cranial nerve is better on 3D-SPACE images (arrows). Axial T1-weighted postcontrast image (c) shows enhancement of the cerebellopontine angle lesion (curved arrow).

**Figure 2:**
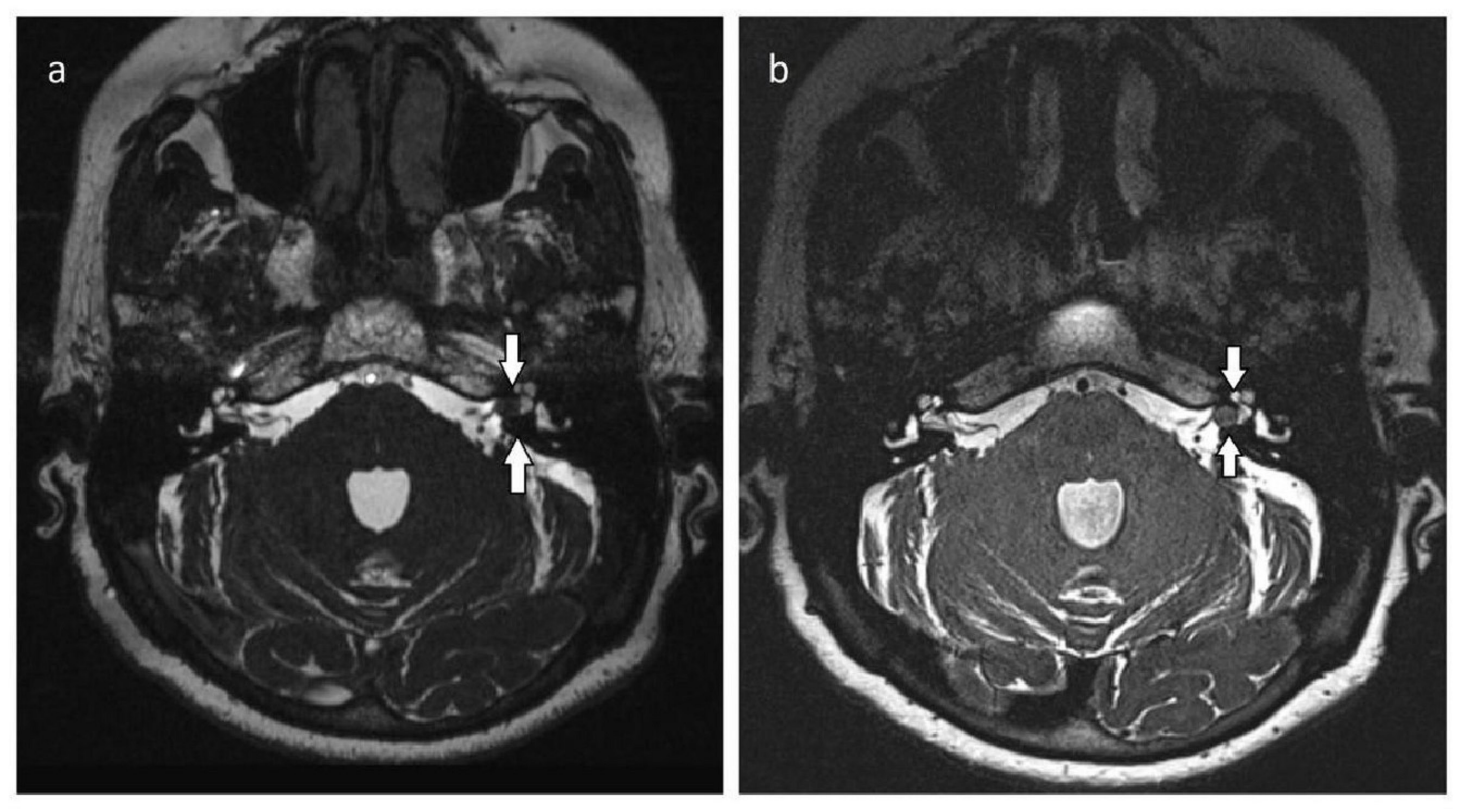
Axial T2-weighted 3D-CISS (a) and (b) 3D-SPACE images of vestibular schwannoma within the left internal auditory canal. Comparison of 3D-CISS and 3D-SPACE sequences. The border between the lesion and adjacent bone is more clearly visualized with 3D-SPACE images (arrows).

**Figure 3:**
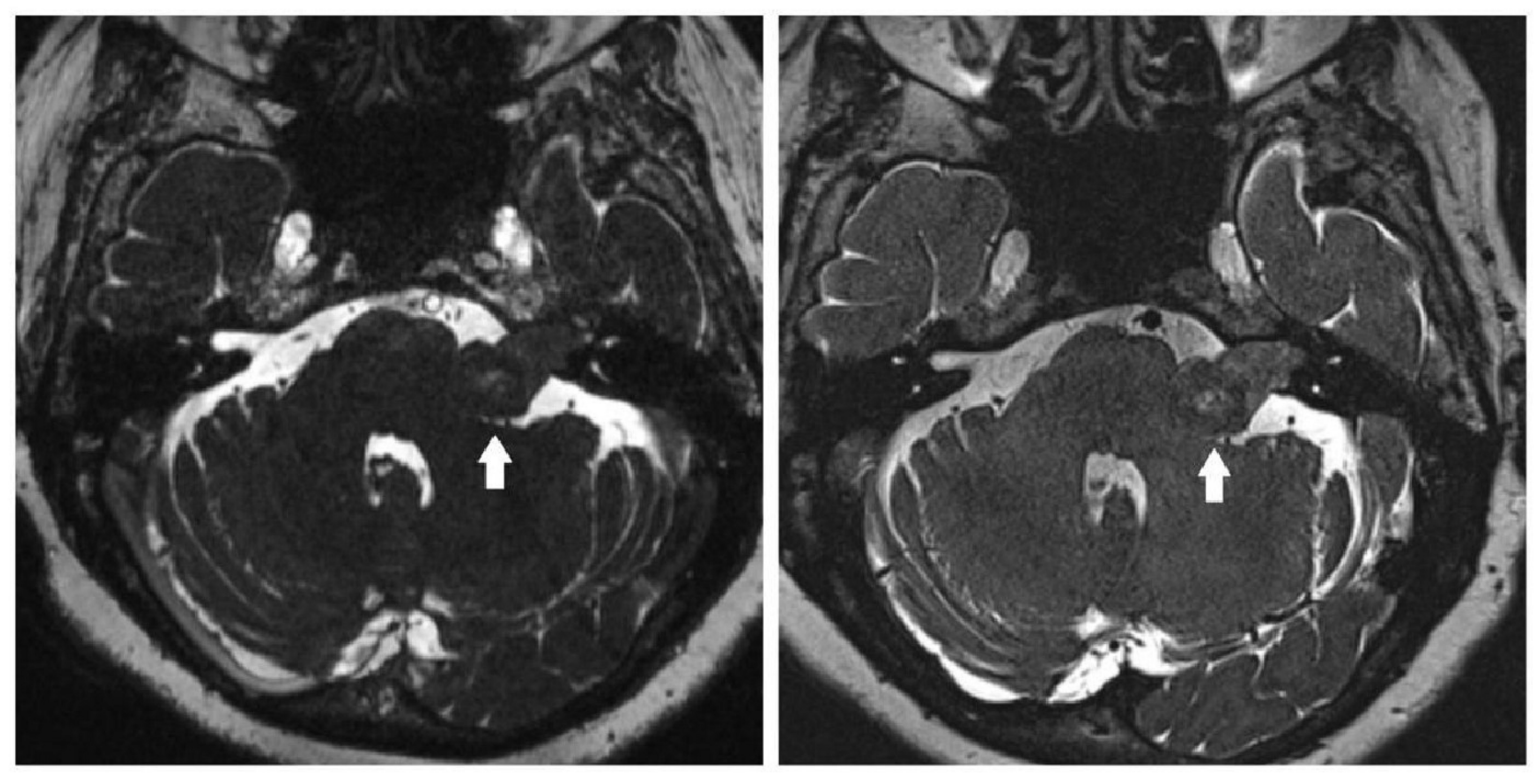
Axial T2-weighted 3D-CISS (a) and (b) 3D-SPACE images demonstrate left cerebellopontine angle schwannoma extending into the internal auditory canal. The signal quality of the lesion and indentation of the adjacent parenchyma is more pronounced on the 3D-SPACE sequences than the 3D-CISS sequences (arrows).

According to the qualitative evaluations of both observers, 3D-SPACE had significantly higher image scores than 3D-CISS in the description of the relationship between lesion and CN, overall image quality, signal difference between the lesion and adjacent brain parenchyma, and signal differentiation between lesion and cistern (p < 0.05). Additionally, 3D-CISS demonstrated more susceptibility and vessel flow artifacts compared to 3D-SPACE (p < 0.05) (Table 4). Illustrative qualitative images for 3D-CISS and 3D-T2-SPACE are shown in Figures 1a, 1b, 1c, 2a, 2b, 3a, and 3b.

**Table 4:** Results of qualitative analyses of two different types of two techniques (*SPACE sampling perfection with application-optimized contrast with different flip-angle evolutions, CISS constructive interference in the steady-state).

		3D-SPACE	3D-CISS	P-value
Overall image quality	Observer 1	3.87 ± 0.335	3.55 ± 0.504	0.001
	Observer 2	3.78 ± 0.423	3.47 ± 0.506	0.006
Visibility of the relationship between the schwannoma and CN	Observer 1	3.53 ± 0.599	2.45 ± 0.783	< 0.001
	Observer 2	3.45 ± 0.597	2.35 ± 0.700	< 0.001
Signal differentiation between lesion and cistern	Observer 1	3.80 ± 0.405	2.70 ± 0.516	< 0.001
	Observer 2	3.70 ± 0.464	2.75 ± 0.543	< 0.001
Signal differentiation between the lesion and adjacent brain parenchyma	Observer 1	3.73 ± 0.452	2.42 ± 0.636	< 0.001
	Observer 2	3.58 ± 0.501	2.33 ± 0.616	< 0.001
Artifacts	Observer 1	2.95 ± 0.389	3.25 ± 0.543	0.006
	Observer 2	2.83 ± 0.446	3.33 ± 0.572	< 0.001

## 4. Discussion

Cerebellopontine angle schwannomas are benign tumors originating from schwann cells and are the most common tumor in this region [3]. CTs and MRIs are the most commonly used imaging methods to evaluate lesions. The main goal of MRI is to determine the association between schwannoma and cranial nerves as well as adjacent brain parenchyma. 3D-SPACE and 3D-CISS can provide more detailed information about CPA with high-resolution images. Furthermore, these sequences are clinically important as they better show the relationship between the lesion with the cranial nerve and the relationship between the lesion and neighboring brain parenchyma.

Haque et al.[13] compared the conventional MRI sequences and histopathological findings in the evaluation of acoustic schwannoma. They reported the sensitivity of MRI to acoustic schwannoma diagnosis as 96%, its specificity as 88.2%, its positive predictive value as 92.31%, and its accuracy as 92.86%.

Abele et al.[14] reported that the combination of non-contrast axial 3D-CISS and coronal T2WI sequences detected schwannomas of less than 10 mm of less than 10 mm in length in the internal acoustic canal (IAC) with 100% accuracy and excellent inter-observer reliability. In our study, the relationship between CPA schwannoma and the cranial nerve was better detected in 3D-SPACE (p < 0.05). Also, 3D-SPACE was superior to 3D-CISS for the evaluation of image quality, signal differences between the lesion and adjacent brain parenchyma (p < 0.05). However, we did not find any significant difference in signal differences between the lesion and adjacent cistern in both sequences (p > 0.05). Tong et al.[15] reported that 3D-SPACE is better than 3D CISS in demonstrating cavernous sinus infestation due to pituitary adenoma. They also concluded that the relationship between the internal carotid artery and the pituitary adenoma was better demonstrated by 3D-SPACE than 3D-CISS. Furthermore, they reported that there were more susceptibility artifacts and vessel flow artifacts in 3D-SPACE than in 3D-CISS. In the same study, they evaluated cavernous sinus invasion using contrast-enhanced T1-weighted 3D-SPACE and CISS sequences. Our study differs in that it demonstrates the relationship between cranial nerve and schwannoma using T2-weighted 3D-SPACE sequences and CISS sequences without the need for contrast media. Watanabe et al. used 3D-CISS and 3D-SPACE sequences to evaluate distal dural ring sign, which is used as an anatomical landmark to distinguish intradural and extradural aneurysms from each other. They reported that 3D-SPACE was superior to CISS for the evaluation of this finding [16]. 

In our study, although both 3D-CISS and 3D-SPACE showed the relationship between the lesion with the cranial nerve and the signal difference between the lesion with the adjacent brain parenchyma, we believe that 3D-SPACE may be more useful for preoperative evaluation. In several studies, it has been reported that 3D-CISS examination is better than T1-weighted images at providing anatomical detail and showing the relationship between the lesion and cranial nerve as well as posterior meatal lip with labyrinth. For this reason, the intralabyrinthine signal intensity in preoperative 3D-CISS has been reported to be an important prognostic marker for predicting postoperative hearing loss [17,18]. 

There were some limitations in our study. Firstly, the number of cases was limited. Secondly, between the two-imaging series, the acquisition time and some of the imaging parameters were not the same and we did not evaluate the diagnostic assessment. In the present analysis, with regards to the output of the same scanning parameters, we suggest that future researches using the same imaging parameters consider some further analysis. Thirdly, the findings of the cases were evaluated only radiologically and there were no surgical or pathological correlations.

In conclusion, T2 weighted-3D-SPACE may be preferred instead of T2 weighted-3D-CISS as CPA as this is more successful for evaluating schwannomas, obtaining better image quality, fewer artifacts, the lesion relationship with the cranial nerve, the adjacent cistern relationship with the lesion, and the adjacent brain parenchyma relationship with the lesion.

## Funding

The authors received no specific funding for this work.

## Ethical approval

The study was approved by the local ethics committee (38-13.01.2020). All procedures performed in studies involving human participants were in accordance with the ethical standards of the institutional research committee and with the 1964 Helsinki Declaration and its later amendments or comparable ethical standards. 

## Informed consent

This is a retrospective study, so informed consent and approval requirement were waived.
